# Betulin Is a Potent Anti-Tumor Agent that Is Enhanced by Cholesterol

**DOI:** 10.1371/journal.pone.0005361

**Published:** 2009-04-28

**Authors:** Franziska B. Mullauer, Jan H. Kessler, Jan Paul Medema

**Affiliations:** 1 Laboratory for Experimental Oncology and Radiobiology (LEXOR), Center for Experimental and Molecular Medicine, Academic Medical Center (AMC), Amsterdam, The Netherlands; 2 Department of Immunohematology and Blood Transfusion, Leiden University Medical Center (LUMC), Leiden, The Netherlands; Ordway Research Institute, United States of America

## Abstract

Betulinic Acid (BetA) and its derivatives have been extensively studied in the past for their anti-tumor effects, but relatively little is known about its precursor Betulin (BE). We found that BE induces apoptosis utilizing a similar mechanism as BetA and is prevented by cyclosporin A (CsA). BE induces cell death more rapidly as compared to BetA, but to achieve similar amounts of cell death a considerably higher concentration of BE is needed. Interestingly, we observed that cholesterol sensitized cells to BE-induced apoptosis, while there was no effect of cholesterol when combined with BetA. Despite the significantly enhanced cytotoxicity, the mode of cell death was not changed as CsA completely abrogated cell death. These results indicate that BE has potent anti-tumor activity especially in combination with cholesterol.

## Introduction

Triterpenoids are extensively studied for the potential use as anticancer agents. One of the most promising compounds in this class is Betulinic Acid (BetA), but its effect is limited by the poor solubility of the compound. A lot of effort is therefore put into the development of derivatives of BetA with the goal to develop even more powerful compounds and to achieve better solubility for enhanced in vivo administration [1]–[3]. BetA has been modified at many different positions including C1-4, C-20, C-28 and A-, D- and E ring with different outcomes [2], [4]. For example, Kvasanica et al found 3beta-O-phthalic esters from BetA more cytotoxic and polar in comparison to BetA itself [5]. In contrast, generation of different C-28 ester derivatives did not result in enhanced cytotoxicity [4]. On the other hand, C-28 amino acid conjugates made by Jeong et al showed improved selective toxicity and solubility [6] and a C-3 modified BetA derivative has shown promising results in a human colon cancer xenograft model [2].

BetA can be found in numerous different plants, but it can also be obtained by a simple 2 step reaction from its more abundantly available precursor molecule Betulin (BE) [3]. BE is easily isolated and therefore plays an important role as raw material for the production of BetA and other biologically active compounds [7]. BE itself has been shown in the past to only possess limited or no cytotoxic effects on cancer cells [5], [8]. For example it was shown to be inactive against MEL-2 (melanoma) cells when compared to other BetA derivatives [9]. Several other melanoma lines (G361, SK-MEL-28) leukemia lines (HL60, U937, K562), and neuroblastoma (GOTO, NB-1) cell lines were also found to be more resistant to BE than to other tested lupane triterpenes [10]. In contrast, a recent report found BE to be active against colorectal (DLD-1), breast (MCF7), prostate ( PC-3) and lung (A549 ) cancer cell lines [11], and for A549 it was shown that apoptosis was induced [12]. Apoptosis is one of the major cell death pathways induced by anti tumor agents. In principle, two main pathways can be distinguished, the extrinsic or death receptor pathway and the intrinsic or mitochondrial pathway with the latter being regulated by the Bcl-2 family of proteins [13]. Numerous studies have shown that BetA induces apoptosis via the mitochondrial pathway [14]–[17], however, to our knowledge, it is currently not clear how BE induces cell death. Here we show that apoptosis induction by BE does not involve the death receptor pathway, but is dependent on the mitochondria. Nevertheless, similar as we have previously shown for BetA [17], cytochrome c release and caspase activation occur independently of the Bcl-2 family proteins but are blocked in the presence of cyclosporin A (CsA), an inhibitor of the mitochondrial permeability transition (PT) pore. Furthermore we found that cholesterol strongly enhances the cytotoxic effects induced by BE but not BetA. Our results suggest that BE should not be regarded as an inactive precursor, but as a potent anti-tumor agent.

## Materials and Methods

### Chemicals

Betulin (≥98% pure; Sigma-Aldrich, St Louis, MO, USA) and Betulinic Acid (≥99% pure; BioSolutions Halle, Germany) were dissolved in DMSO at 4 mg/ml, cholesterol (Sigma-Aldrich) was dissolved at 5 mM in DMSO. Aliquots were kept frozen. Propidium iodide (PI), zVAD.fmk (benzyloxycarbonyl-Val-Ala-Asp-fluoromethylketone), etoposide and cyclosporin A were purchased from Sigma-Aldrich, Mitosox was obtained from Invitrogen (Carlsbad, CA, USA).

### Antibodies

Anti-PARP (#9542; Cell Signaling Technology, Danvers, MA, USA) and anti-cytochrome c (clone 6H2.B4; BD Biosciences, San Diego, CA, USA) were used.

*Cell lines*: A549 and Hela were obtained from the ATCC, FADD-deficient, Caspase 8- deficient and control Jurkat cells (JA3) were kindly provided by Dr John Blenis (Harvard Medical School, Boston), Jurkat cells over-expressing Bcl-2 by Dr Jannie Borst (NKI, Amsterdam) and Bax/Bak double knockout (DKO) mouse embryonic fibroblasts (MEFs) and wild-type control MEFs were from Dr Stanley Korsmeyer.

### Cell death analysis

Overall cell death was assessed as previously described [18] by PI exclusion assay. Briefly, cells were incubated with 1 µg/ml PI and measured by flow cytometry.

### DNA fragmentation

Cells were incubated in Nicoletti buffer containing 50 µg/ml PI for at least 24 hours before analysis via flow cytometry.

### Western blot analysis (immunoblotting)

Cells were lysed using Triton X-100 buffer and for protein quantification a BCA kit from PIERCE was used. SDS-PAGE was performed and proteins were transferred onto a PVDF transfer membrane (Amersham Biosciences). Blocking of unspecific binding sites was achieved by incubation of the membrane in 5% low fat milk powder in PBS/0.2% Tween-20 (blocking buffer) for 1 hour at room temperature. Primary antibody incubation was performed overnight at 4°C and secondary antibody (HRP labeled) incubation for 2 hours at room temperature. For chemiluminescent detection ECL from Amersham Biosciences was used in combination with a LAS-3000 imaging system.

### ROS detection

For ROS measurements the highly selective dye for mitochondrial superoxide Mitosox was used. Cells were incubated with 5 µM Mitosox in pre-warmed tissue culture medium at 37°C for 10 min before flow cytometry analysis.

### Cytochrome c release by FACS staining

Cytochrome c release was measured as previously described by Waterhouse et al [19]. First, outer cell membrane permeabilization was achieved by incubation for 5–10 minutes with 50 µg/ml digitonin in PBS containing 100 mM KCl. Cells were then fixed in 4% paraformaldehyde for 30 minutes at room temperature, washed and incubated in blocking buffer (3% BSA, 0.05% saponin, 0.02% azide in PBS supplemented with normal goat serum, dilution 1:200). Anti cytochrome c incubation was done overnight at 4°C and for flow cytometric detection a FITC conjugated secondary antibody was applied.

### MTT assay

cells were incubated in the presence of 40 µg/ml MTT reagent for 2 hours at 37°C. During the incubation period appearance of purple formazan structures was followed by phase-contrast light microscopy.

## Results

### Cholesterol strongly enhances cytotoxic effects of BE but not BetA

**Figure 1:**
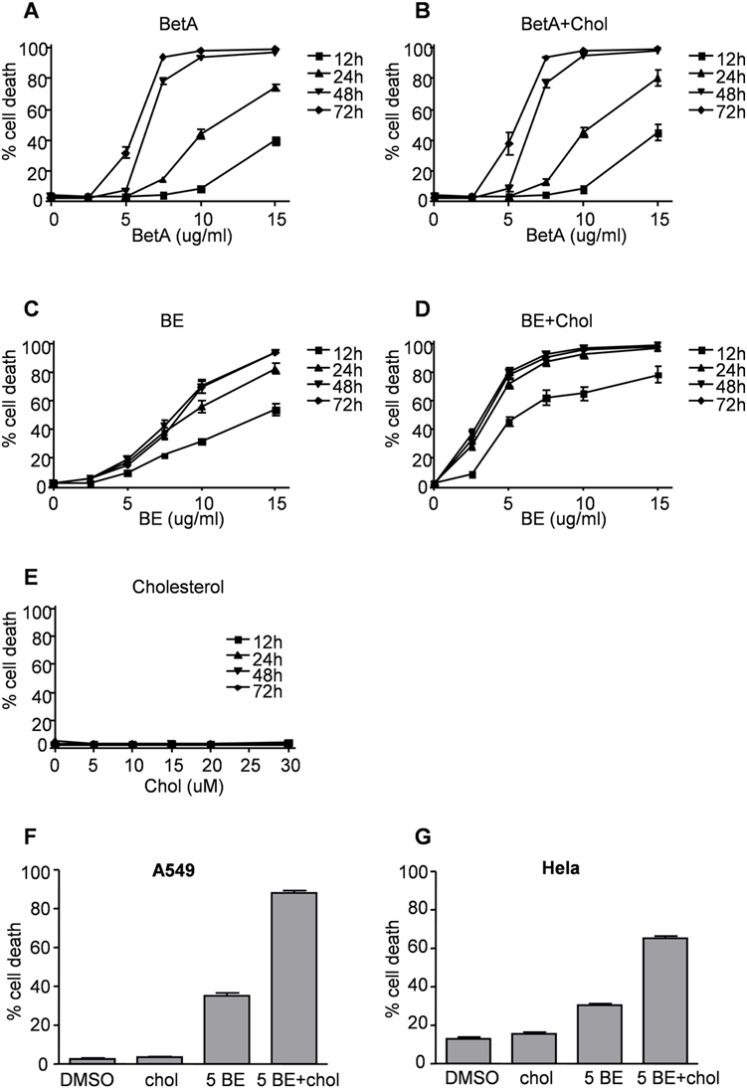
Cholesterol strongly enhances cytotoxic effects of BE but not BetA. Jurkat cells were treated with the indicated concentrations of BetA (A), BetA in combination with 5 µM cholesterol (B), BE (C), BE in combination with 5 µM cholesterol (D) or various concentrations of cholesterol only (E). Cell death was monitored after 12, 24, 48 and 72 hours using PI exclusion. A549 lung cancer (F) and HeLa cervix carcinoma (G) cell lines were treated with 5 µM cholesterol (chol), 5 µg/ml BE (5 BE) or the combination of 5 µg/ml BE with 5 µM cholesterol (5 BE+chol) and after 24 hours cell death was analyzed via PI exclusion.

Previously we have shown that BetA induces cell death in Jurkat T leukemia cells in a concentration and time-dependent fashion [18]. Here we show that low concentrations (5 µg/ml) of BetA are non toxic up to 48 hours incubation and show limited cell death after 72 hours (Figure 1A). In contrast, when 7.5 µg/ml BetA or more is used almost all cells are PI positive after 48 to 72 hours (Figure 1A). To analyze whether Betulin (BE), the precursor of BetA, is capable of inducing cell death we titrated BE on Jurkat T Leukemia cells. In contrast to previous reports we show here that BE is capable of killing cells, but required higher concentrations than BetA. However, it appeared that cell death induced by BE is more efficient after 12 hours when compared to BetA and maximum cell death is achieved after 24 hours (Figure 1C).

We have found previously that when using the MTT (3-(4,5-Dimethylthiazol-2-yl)-2,5-diphenyltetrazolium bromide) assay to measure BetA [18] or BE (unpublished data) induced cytotoxicity, results were much more pronounced when compared to other assays such as PI exclusion and clonogenic survival [18]. This decrease in MTT conversion is likely the result of a direct effect of BetA on the mitochondria and was accompanied by a different morphological appearance of the formazan precipitates. While normal formazan formation shows a punctuate appearance, BetA and BE-induced formazan formation shows the rapid appearance of needle-like structures on the cell surface (Figure S1). Interestingly, cholesterol, which shares some structural similarities with BE and BetA, has been reported to have a comparable effect in the MTT assay [20]–[22] (Figure S1). This suggests that cholesterol, BetA and BE may share common targets in the cell. To clarify if this feature is related to the cytotoxicity of these compounds we decided to analyze the effect of cholesterol on cell death and combine cholesterol with either BetA or BE and measure PI exclusion after various time points. Cholesterol itself did not induce cell death in Jurkat cells (Figure 1E) and it did not enhance cytotoxicity of BetA at all time points measured (Figure 1B). However, the combination of BE with cholesterol resulted in massive cell death in Jurkat cells even when very small concentrations of BE were used (2.5 and 5 µg/ml BE, Figure 1D). To rule out that this is a cell type specific effect we analyzed cell death in A549 (lung carcinoma) and HeLa (cervical carcinoma) cells exposed to either BE or BE in combination with cholesterol. Similar to what was observed with the Jurkat cells, both solid cancer cell lines displayed massive cell death when treated with the combination of BE and cholesterol, whereas BE by itself showed only minor toxicity at the concentration used (Figure 1F and 1G).

### BE/Cholesterol induces apoptosis in Jurkat cells

**Figure 2:**
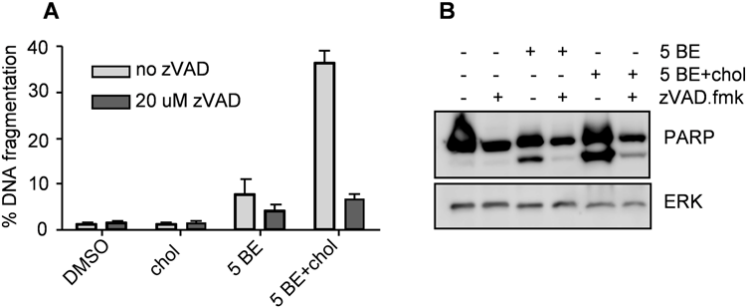
BE/cholesterol induces apoptosis in Jurkat cells. (A) Jurkat cells were pretreated with 20 µM zVAD.fmk for at least one hour prior to addition of either DMSO, 5 µM cholesterol (5 Chol), 5 µg/ml BE (5 BE) or 5 µg/ml BE in combination with 5 µM cholesterol (5 BE+chol). After 24 hours DNA fragmentation was assessed by FACS analysis of propidium iodide (PI) stained nuclei. (B) Jurkat cells were treated as described in (A) but after 24 hours PARP cleavage was assessed by immunoblotting. The protein kinase ERK was used as a loading control.

To identify the nature of cell death induced by BE/Cholesterol we investigated the apoptotic pathway. Apoptosis has been previously reported to be the cell death pathway induced by BE in A549 lung cancer cells [12]. We assessed DNA fragmentation as an apoptosis read-out in Jurkat cells treated for 24 hours with either cholesterol, BE or the combination of both. In cells treated with cholesterol only, DNA fragmentation was completely absent (Figure 2A), consistent with the lack of cell death. BE, at 5 µg/ml, induced only moderate DNA fragmentation. However, when combined with cholesterol DNA was clearly fragmented (Figure 2A). To verify these results we performed immunoblotting for the classical caspase target PARP and observed similar effects: Upon BE treatment PARP was processed to some extent and this was strongly enhanced by addition of cholesterol (Figure 2B). Importantly, both, DNA fragmentation and PARP cleavage were blocked when cells were pre-treated with zVAD.fmk (a pan-caspase inhibitor) confirming that both are caspase-mediated events (Figure 2A and 2B).

### The death receptor pathway is not involved in BE/cholesterol induced apoptosis

**Figure 3:**
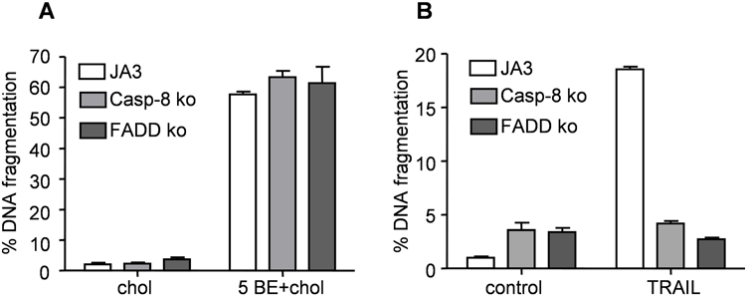
The death receptor pathway is not involved in BE/cholesterol induced apoptosis. Jurkat control (JA3), FADD deficient (FADD ko) or caspase-8 deficient (Casp-8 ko) cells were treated with TRAIL (0.5 µg/ml plus 1 µg/ml anti-FLAG) (A) or with either 5 µM cholesterol (chol) or 5 µg/ml BE in combination with 5 µM cholesterol (5 BE+chol) and after 24 hours DNA fragmentation was analyzed.

Cholesterol is an important constituent of cell membranes where it plays a crucial role in maintaining integrity and fluidity [23]. In addition, cholesterol-enriched micro-domains, so called lipid rafts, are important signal transduction platforms [24], which have been related to apoptosis [25] and changes in plasma cholesterol levels have been associated with Fas-FADD complex formation and caspase-8 activation [26], [27]. BetA has been shown to induce apoptosis independently of the extrinsic pathway [28]. However, because of the strong apoptosis-enhancing effects of cholesterol when combined with BE, we decided to investigate the involvement of this pathway by applying BE/cholesterol on Jurkat cells either deficient for FADD or caspase-8. Recently we showed that the FADD and caspase-8 deficient cells were completely resistant to Fas-induced apoptosis [17]. Here this resistance was further confirmed using TRAIL (Figure 3A). Despite the resistance towards the extrinsic pathway, neither cell line showed decreased DNA fragmentation when treated with BE/cholesterol (Figure 3B), indicating that the death receptor pathway is not involved in BE/cholesterol-induced apoptosis.

### BE/cholesterol induced apoptosis is mechanistically related to BetA induced apoptosis

BetA induced apoptosis has been clearly linked to the mitochondria [14]–[17] with the consistently described features of cytochrome c release and induction of reactive oxygen species (ROS) [28]–[31]. These events were initially described to be Bcl-2 family dependent [15], [16], however, our recent evidence suggests only a minor role for the Bcl-2 family proteins. Instead we proposed a direct effect on the PT-pore [17]. To test if BE/cholesterol induces apoptosis via similar mechanisms as BetA we investigated the mitochondrial pathway of apoptosis.

**Figure 4:**
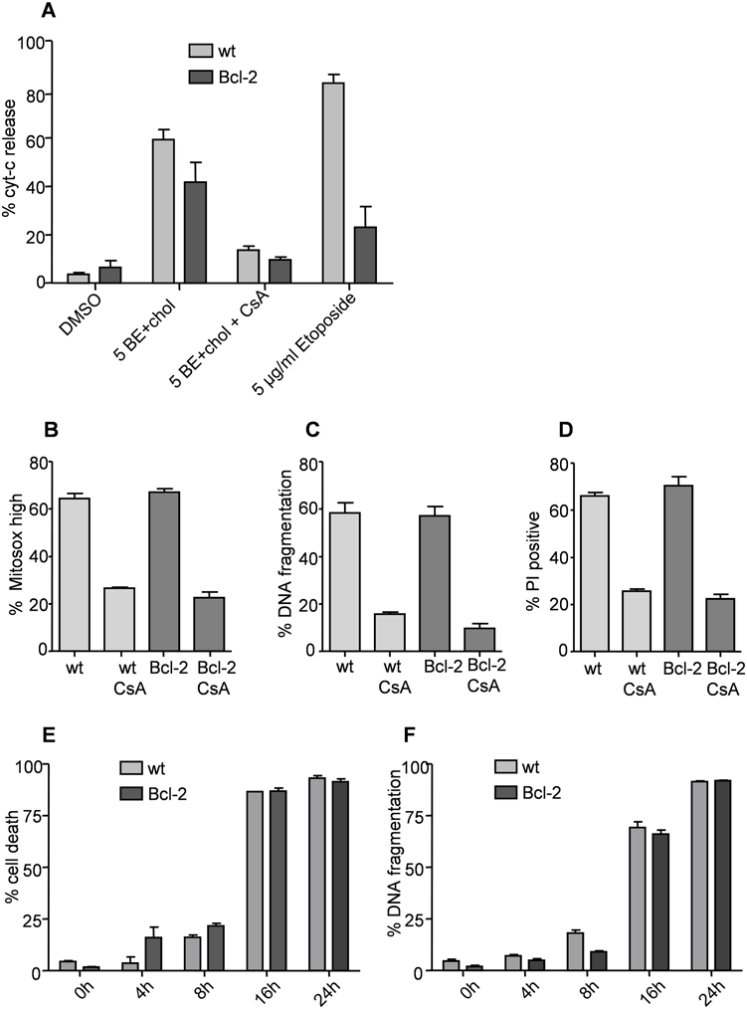
BE/cholesterol induced apoptosis is not affected by Bcl-2 over-expression but is inhibited in the presence of cyclosporin A. (A) Jurkat control (wt) or Bcl-2 over-expressing cells (Bcl-2) were treated as indicated (5BE = 5 µg/ml BE; chol = 5 µM cholesterol; CsA = 5 µg/ml cyclosporin A), after 24 hours intracellular staining for cytochrome *c* release was performed. (B, C, D) Jurkat control (wt) or Bcl-2 over-expressing cells were treated with 5 µg/ml BE/ 5 µM cholesterol either in the absence or presence of 5 µg/ml cyclosporin A. After 24 hours ROS (B), DNA fragmentation (C) and overall cell death (D) were assessed by FACS analysis. (E, F) Jurkat control (wt) or Bcl-2 over-expressing cells were treated with 5 µg/ml BE/ 5 µM cholesterol and PI exclusion (E) or DNA fragmentation (F) were measured after 0, 4, 8, 16 and 24 hours.

BE/cholesterol showed clear cytochrome c release in Jurkat cells. Importantly, there was only a slight difference in cytochrome c release in the Bcl-2 over-expressing cells (Figure 4A), but this difference was statistically not significant (paired t-test). Jurkat cells over-expressing Bcl-2 were completely resistant to etoposide (Figure. 4A). In contrast to the lack of effect of Bcl-2 over-expression, CsA provided almost complete protection (Figure 4A). To determine if ROS are produced upon BE/cholesterol treatment we used a dye specifically detecting mitochondrial superoxide. Both wildtype as well as Bcl-2 over-expressing cells showed clear increase in ROS, strikingly this was again abolished in the presence of CsA (Figure 4B). To verify that these events resemble the amount of apoptosis and overall cell death we measured DNA fragmentation and PI exclusion respectively. Bcl-2 over-expression did not provide any protection whereas CsA effectively prevented both, apoptosis and cell death (Figure 4C and 4D). In order to find out if Bcl-2 over-expression causes a delay in apoptosis as is the case with BetA [17], we performed a kinetic analysis. Cell death and DNA fragmentation were measured after various time points from 0–24 hours. At all time points we did not observe any difference in sensitivity to BE/cholesterol, further underscoring the lack of inhibition by Bcl-2 (Figure 4E and 4F). These results suggest that BE/cholesterol kills Jurkat cells by inducing mitochondrial damage that leads to cytochrome c release and apoptosis which is completely independent of Bcl-2.

**Figure 5:**
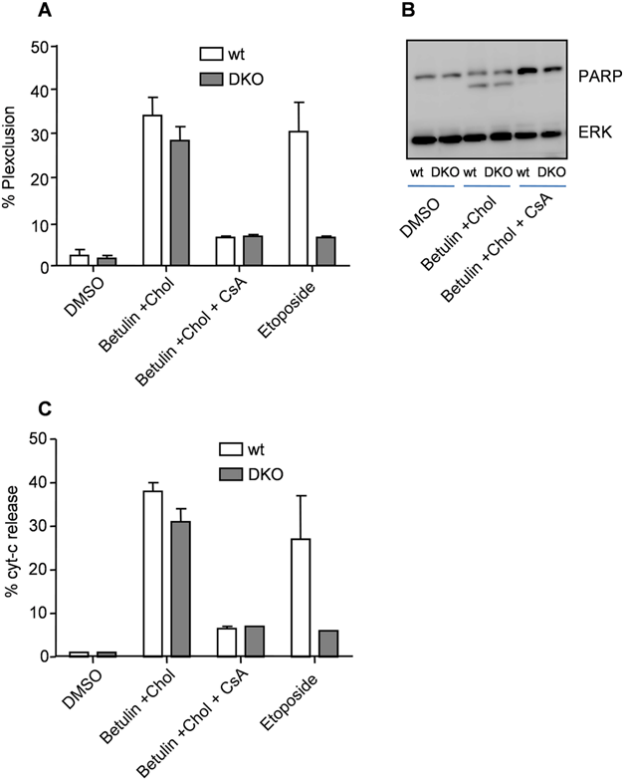
BE/cholesterol induced apoptosis is independent of Bax/Bak. (A) Wildtype (wt) or Bax/Bak double knockout (DKO) mouse embryonic fibroblasts (MEFs) were treated as indicated and after 24 hours cell death was assessed by PI exclusion. Etoposide was included as a control for functionality of the cells. (B) Wt or DKO MEFs were treated as indicated and after 24 hours cells were subjected to immunoblotting to determine PARP processing. ERK was used as control for equal protein amounts. (C) Wt and DKO MEFs were treated as indicated for 24 hours before measuring cytochrome *c* release by intracellular FACS staining.

To further determine the efficacy of BE/cholesterol and to find out if Bax and Bak are involved in BE/cholesterol induced cytotoxicity we used Bax/Bak double-knockout (DKO) mouse embryonic fibroblasts (MEFs). DKO MEFs are resistant to drugs such as etoposide, staurosporine, UVC or actinomycin D, all targeting the Bcl-2 family regulated mitochondrial pathway [32]. We measured PI exclusion and found DKO MEFs to be sensitive to BE/cholesterol, as a control for the functionality of the cells etoposide was included (Figure 5A). We assessed if apoptosis was induced like in BetA treated cells by analyzing PARP cleavage. PARP was clearly processed in wildtype as well as in DKO MEFs, suggesting that Bax and Bak are not essential in BE/cholesterol induced apoptosis (Figure 5B). Also cytochrome c release was not prevented in DKO MEFs (Figure 5C), further substantiating that Bax and Bak are not required for BE/cholesterol mediated cytotoxicity. Similar to Jurkat cells, CsA provided complete protection against cell death (Figure 5A), apoptosis (Figure 5B) and cytochrome c release (Figure 5C), confirming the crucial role for the mitochondrial permeability transition in BE/cholesterol induced cytotoxicity.

## Discussion

BE is a natural compound, which contains derivatives that have been shown to possess strong anti-tumor properties [7], [33]. Here we provide evidence that BE itself, especially in combination with cholesterol (BE/cholesterol), is very potent in killing cancer cells in vitro (Figure 1). BE/cholesterol induces apoptosis in a similar manner as BetA and does not involve the extrinsic pathway of apoptosis (Figure 3), but instead apoptosis depends on the mitochondrial pathway (Figure 4). However, as we reported for BetA, this pathway is activated in an unconventional manner as cytochrome c release and apoptosis are induced in cells over-expressing Bcl-2 (Figure 4) or in cells deficient for Bax/Bak (Figure 5), while both events are blocked by CsA (Figure 4 and Figure 5). This indicates that permeability transition is pivotal in the process of BE/cholesterol induced cytotoxicity.

Despite the strong similarities, and the almost identical structure of BE and BetA, there are also important differences in comparison to BetA induced apoptosis. We previously showed that Bcl-2 over-expression delayed BetA-induced apoptosis[17], but curiously in the case of BE/cholesterol it has very limited effect on the amount of cytotoxicity induced (Figure 4). Furthermore, CsA by itself provides much stronger protection in the case of BE/cholesterol in Jurkat cells, while BetA treated Jurkat cells are only completely protected when a combination of CsA with Bcl-2 over-expression is used.

This difference between BetA and BE/cholesterol is even more remarkable when considering the time dependency of cytotoxicity of both molecules: For BetA the maximum effect requires around 48–72 hours and a dose of 7.5–10 µg/ml (Figure 1A and 1B), while BE/cholesterol induced death is already maximum at 24 hours. Nevertheless, CsA is capable of providing efficient protection.

Striking is the fact, that cholesterol strongly enhances the cytotoxic effects of BE but not BetA (Figure 1B and 1D) whilst being completely non-toxic on its own, even at very high concentrations (Figure 1E). Currently we do not know the mechanism by which cholesterol acts as a “cytotoxicity-amplifier” for BE but it likely involves membrane integrity. Cholesterol is abundantly present in the plasmamembrane and it is possible that changes in cholesterol content can affect the amount of BE that is taken up by a cell.

The effect on MTT conversion to formazan (MTT measures mitochondrial enzymatic activity [20], [34]) by all three compounds, BetA, BE and cholesterol, suggests a common target in the mitochondria. Even though this is clearly not directly related to cytotoxicity, as cholesterol on its own is completely non-toxic, it may point to a mechanism that sensitizes cells to BE. It is not clear how this is orchestrated but it could involve the mitochondrial membrane, for instance mitochondrial PT pore opening. The exact composition of the pore has yet to be established but adenine-nucleotide-translocator (ANT), voltage-dependent-anion-channel (VDAC) and cyclophilin D are discussed as core components in the currently accepted model [35]. PT pore opening is influenced by the amount of cholesterol present in the mitochondrial membrane, cholesterol affects VDAC function [35] and impairs ANT mediated PT through altered membrane fluidity [36]. So cholesterol-induced effects on the PT pore may facilitate BE-induced opening. Why this then does not influence BetA-induced opening is unclear at this point and will require further investigation. In this light it is also important to realize that Bcl-2 over-expression delays BetA-induced apoptosis [17], while CsA can only partially prevent the induction of apoptosis. This suggests that BetA may has a direct effect on the PT pore, which is blocked by CsA and maybe also induces a more classical Bcl-2-dependent pathway to cytochrome c release. This latter seems absent when using BE and may be the reason these compounds react slightly different to CsA and potentially also cholesterol.

To further evaluate the anti-tumor properties of BE/cholesterol *in vivo* studies will be required. Preliminary results from a pharmacokinetic study using triterpene extract (TE) mainly consisting of Betulin suggest that it is safe; no signs of toxicity were observed in rats or dogs in a subchronic toxicity study [37]. Another study investigated the effects of BE on the central nervous system (CNS) with the conclusion that there was no effect of BE on muscle tone and coordination in mice; doses up to 100 mg/kg bodyweight were used [38]. Interestingly another study explored the antinociceptive properties of Betulin in mice and results suggest that it is even more active than aspirin and paracetamol [39].

It will be interesting to explore the combined effects of BE and cholesterol *in vivo*. Because cholesterol is ubiquitously present in the body it is unlikely that additional applied cholesterol is useful for *in vivo* effects of BE as an anti-tumor agent. Our results indicate that the amount of cholesterol necessary (5 µM) for enhanced *in vitro* effects of BE are about 1000 times lower than normal plasma cholesterol levels in humans (5 mM). However the fast majority of this cholesterol is contained in LDL or HDL and it is therefore difficult to assess whether there is sufficient free cholesterol available to potentiate BE-induced apoptosis in vivo. Adding more cholesterol may not bear any significance though, but application of cholesterol containing Betulin-liposomes may be an interesting mode of applying this cytotoxic agent. In summary we conclude that Betulin by itself and in combination with cholesterol is a potent anti-cancer agent *in vitro* and warrants further investigation *in vivo*.

## Supporting Information

Figure S1 MTT conversion. Effects of BetA, cholesterol and BE on MTT assay: Jurkat cells were treated as indicated, incubated with MTT reagent and photographed under a phase-contrast microscope.
(6.51 MB TIF)
