# Supplementary figures and images for: Betulin Is a Potent Anti-Tumor Agent that Is Enhanced by Cholesterol
Source: PLoS One. 2009 Apr 28;4(4):e1. doi: 10.1371/journal.pone.0005361 (PMC2671171; doi:10.1371/journal.pone.0005361)

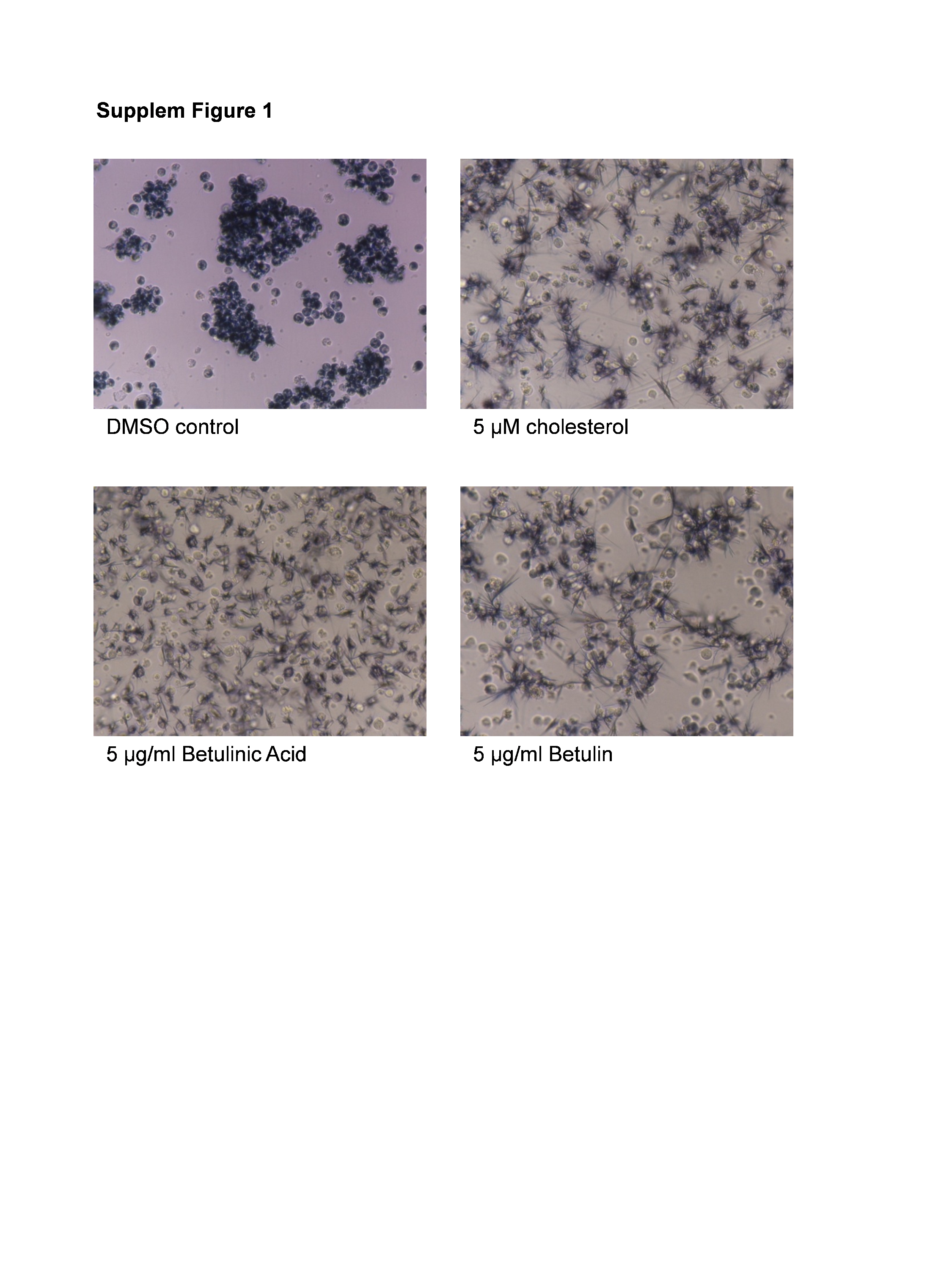

Supplement: Figure S1 — MTT conversion. Effects of BetA, cholesterol and BE on MTT assay: Jurkat cells were treated as indicated, incubated with MTT reagent and photographed under a phase-contrast microscope. (6.51 MB TIF) [file pone.0005361.s001.tif]
